# Bibliometrics and visualization analysis: Big data research on diabetic kidney disease from 2000 to 2025

**DOI:** 10.1097/MD.0000000000050234

**Published:** 2026-08-14

**Authors:** Tingting Ding, Shang Li, Qinglin Guo, Mingkang Zhang, Yazhi Wang

**Affiliations:** a Department of Radiation Oncology, Gansu Provincial Hospital, Lanzhou, Gansu, China; b The First School of Clinical Medicine, Lanzhou University, Lanzhou, Gansu, China; c School of Pharmacy, Lanzhou University, Lanzhou, Gansu, China; d The Second School of Clinical Medicine, Lanzhou University, Lanzhou, Gansu, China.

**Keywords:** artificial intelligence, bibliometrics, big data analysis, diabetic kidney disease, machine learning

## Abstract

**Background:**

As the prevalence of diabetes rises, diabetic kidney disease (DKD) has become a leading cause of end-stage renal disease. Big data analysis aids in DKD prediction, diagnosis, and personalized treatment. This bibliometric study summarizes the current research status and hotspots in big data-driven DKD research.

**Methods:**

On March 26, 2025, DKD-related big data publications were retrieved from the Web of Science Core Collection. CiteSpace and VOSviewer were used for co-authorship, co-occurrence, and co-citation analyses to construct knowledge networks.

**Results:**

Three hundred twenty documents were identified, involving 2176 authors, 695 institutions, and 51 countries/regions, published in 192 journals. Research grew gradually from 2002 to 2018 and then rapidly after 2019. *Frontiers in Endocrinology* (21 publications) and *Journal of the American Society of Nephrology* (421 citations) led in publications and citations, respectively. China (189 publications), Beijing University of Chinese Medicine (10 publications), and Donovan, Michael J (6 publications) were the most productive. Hotspots included DKD (192), machine learning (ML, 99), diabetes mellitus (82), prediction (61), risk (52), chronic kidney disease (51), biomarkers (41), progression (33), artificial intelligence (AI, 27), and expression (27). ML, AI, mechanisms, and cells may be future frontiers.

**Conclusion:**

Big data-driven DKD research is growing, with multi-omics biomarkers underpinning AI/ML models that are hotspots for risk prediction and progression assessment; AI, ML, mechanisms, and cells are the frontiers, which together provide references for DKD precision medicine.

## 1. Introduction

Diabetic kidney disease (DKD), one of the most severe microvascular complications of diabetes, has emerged as the leading cause of end-stage renal disease (ESRD) worldwide.^[1]^ Approximately 20% to 40% of patients with diabetes globally will ultimately develop DKD. The pathogenesis is highly complex, involving multiple pathological pathways such as glucose and lipid metabolism disorders, oxidative stress, inflammatory responses, autophagy, and fibrosis, which can induce glomerulosclerosis, podocyte injury, and renal interstitial fibrosis.^[2]^ Consequently, the onset and progression of DKD exhibit significant heterogeneity. Despite substantial clinical efforts in DKD diagnosis and treatment, which have improved renal function in some patients, early diagnosis, risk assessment, and precise intervention remain critical challenges for healthcare professionals.^[1]^

Big data analysis refers to a technological framework that employs high-performance computing to integrate and analyze complex datasets, thereby extracting hidden insights. It is characterized by the 6V features: volume, velocity, variety, veracity, value, and variability.^[3]^ In recent years, the advent and widespread application of big data analysis in the medical field have significantly enhanced the efficiency and precision of clinical research.

Currently, big data analysis holds significant potential in DKD research. For instance, integrated transcriptomic and proteomic analyses have revealed aberrant activation of key signaling pathways (e.g., umor necrosis factor-alpha/nuclear factor-kappa B and transforming growth factor-beta/mothers against decapentaplegic homolog) in DKD pathogenesis,^[4]^ while identifying multiple potential diagnostic biomarkers such as urinary exosomal microRNAs (miRNAs)^[5]^ and serum inflammatory factors.^[6]^ In clinical practice, predictive models demonstrate strong accuracy for estimating chronic kidney disease (CKD) risk in patients with diabetes, thereby supporting personalized treatment strategies.^[7]^ Furthermore, network pharmacology combined with molecular docking techniques has successfully elucidated drug-target interactions in DKD, accelerating the development of targeted therapies.^[8]^ Similarly, a recent in silico study applied molecular docking and dynamic simulation to identify potential phytocompounds targeting multiple pathways in lupus nephritis, further supporting the value of computational approaches in kidney disease research.^[9]^

Bibliometrics is a scientific methodology for the quantitative analysis of academic publications. Through co-authorship, co-occurrence, co-citation, and cluster analysis, it enables systematic synthesis of research progress in specific fields.^[10]^ Despite extensive research on big data analysis in DKD, a comprehensive bibliometric analysis in this field remains unexplored.^[4–7]^ This study aimed to employ bibliometric analysis coupled with knowledge mapping techniques (CiteSpace/VOSviewer) to systematically characterize big data analysis research in DKD, including journals, publications, countries/regions, institutions, authors, references, and keywords.

## 2. Materials and methods

### 2.1. Data collection and retrieval strategies

We systematically retrieved publications related to big data analysis in DKD from the Web of Science Core Collection (WoSCC) database. The time frame was January 1, 2000, to March 26, 2025. All records were downloaded in plain text format, with the publication timeline spanning from the inception of the database through 2025. The publication year of each record was determined by its formal issuance date. The search strategy was collaboratively developed by the research team. The retrieval process consisted of the following key steps: #1=((((((((TS=(“Diabetic Nephropathy”)) OR TS=(“Nephropathy, Diabetic”)) OR TS=(“Diabetic Kidney Disease”)) OR TS=(“Kidney Disease, Diabetic”)) OR TS=(“Diabetic Kidney Diseases”)) OR TS=(“Kidney Diseases, Diabetic”)) OR TS=(“Diabetic Glomerulosclerosis”)) OR TS=(“Glomerulosclerosis, Diabetic”)) OR TS=(“DKD”); #2=((((((((((((((((((TS=(“Big Data”)) OR TS=(“Big Data Analytics”)) OR TS=(“Large* Data”)) OR TS=(“Data Mining”)) OR TS=(“Data Science”)) OR TS=(“Knowledge Discovery”)) OR TS=(“Knowledge Mining”)) OR TS=(“Information Retrieval”)) OR TS=(“Information Extraction”)) OR TS=(“Deep Learn*”)) OR TS=(“Cloud Comput*”)) OR TS=(“Data Warehous*”)) OR TS=(“Data Librar*”)) OR TS=(“Data Bank*”)) OR TS=(“Machine Learning”)) OR TS=(“Artificial Intelligence”)) OR TS=(“Statistical Learn*”)) OR TS=(“Neural Network*”)) OR TS=(“Predictive Model*”); and #1 AND #2.

### 2.2. Inclusion and exclusion criteria

**Figure 1.**
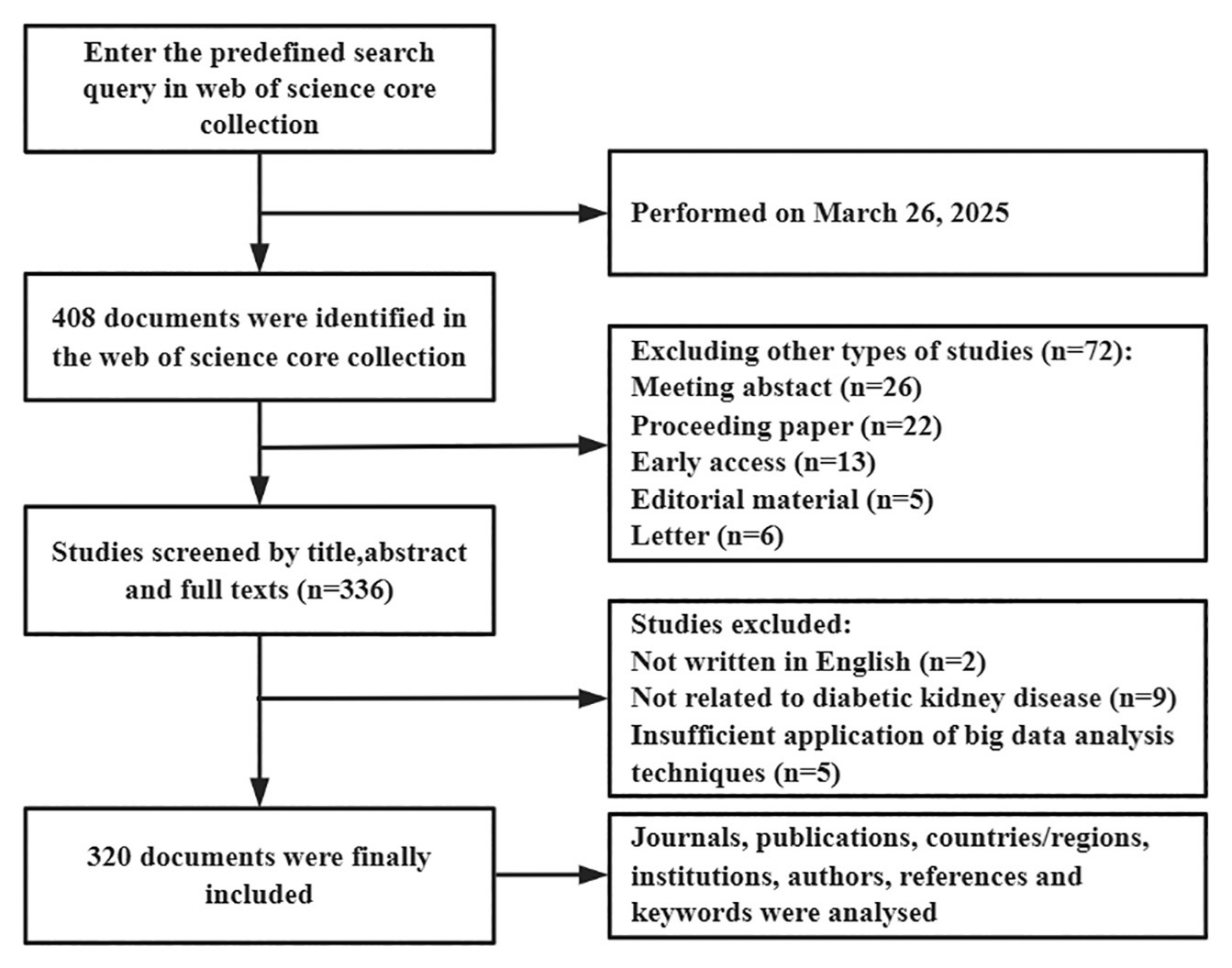
Flowchart of this study.

To ensure that the results of the analysis were in line with the paradigm of “data-driven medical research,”^[11]^ the following criteria were established in this study. Inclusion criteria were as follows. First, the literature should be published in English. Second, the research topic must be directly related to DKD, with specific sub-directions and their respective judgment criteria detailed as follows: Mechanism research: Studies employing big data technologies to explore the pathophysiological mechanisms of DKD. This specifically includes identifying key molecules (genes, proteins, metabolites), signaling pathways, or cellular regulatory networks involved in the initiation and progression of DKD through multi-omics analyses (genomics, transcriptomics, proteomics, metabolomics, lipidomics); conducting bioinformatics analysis of large-scale biological datasets to elucidate the molecular mechanisms of DKD; and employing systems biology approaches to integrate multidimensional data, thereby revealing the complex pathogenic networks of DKD. Disease prediction and risk stratification: Studies utilizing big data methodologies to develop or validate prediction models related to DKD. This specifically includes constructing models to predict the onset, progression (e.g., transition from microalbuminuria to macroalbuminuria), or decline in renal function (e.g., estimated glomerular filtration rate decline ≥30% or progression to ESRD) based on clinical data (electronic health records [EHR], laboratory parameters), imaging data, or biomarkers; applying machine learning (ML), artificial intelligence (AI), or statistical learning techniques to stratify the risk of DKD in diabetic patients and identify high-risk populations; and validating the effectiveness of prediction models in multicenter, large-sample cohorts. Diagnostic and prognostic biomarkers: Studies employing big data technologies to screen, identify, or validate biomarkers related to DKD diagnosis or prognosis. This specifically includes high-throughput screening of potential biomarkers (e.g., urinary exosomal microRNAs, serum inflammatory factors, and proteomic markers) from large-scale samples; validating the specificity, sensitivity, and clinical application value of biomarkers through big data analysis; and developing DKD diagnostic or prognostic tools based on biomarkers. Clinical management and precision therapy: Studies utilizing big data to optimize the clinical management of DKD or explore precision treatment strategies. This specifically includes evaluating the efficacy and safety of therapeutic interventions for DKD (e.g., medications, dialysis, and transplantation) by analyzing large-scale clinical data; applying AI or ML to formulate personalized treatment plans based on individual patient characteristics (genetic background, clinical data, lifestyle factors); and leveraging big data to explore the associations between DKD and its complications (e.g., diabetic retinopathy and cardiovascular diseases [CVD]), thereby providing a basis for comprehensive clinical management. Third, the study must explicitly specify the application of big data analysis methods, such as ML, EHR analysis, multi-omics data analysis, intelligent diagnosis based on medical imaging or sensor data, or multidimensional data analysis using large-scale databases. The application of big data technology must constitute a core methodological component of the study, not merely a superficial mention. Exclusion criteria were as follows: insufficient relevance of the study topic to DKD; the publication types were not “article” and “review,” for example, meeting abstracts and letters; insufficient application of big data technology, for example, the concept of “big data” was only mentioned, but the technology was not applied in practice; and lack of data processing procedures, for example, studies were excluded if they failed to clearly describe the essential data processing steps required for their big-data analysis. Specifically, a study was deemed to have “insufficient data processing procedures” if it met any of the following conditions: missing data preprocessing, meaning the study did not mention or adequately describe at least one key preprocessing step specific to its data type – for example, failing to explain how to handle missing values, outliers, or data normalization for clinical or EHR data; not mentioning background correction, normalization, or batch effect correction for multi-omics data; or not describing basic preprocessing such as image quality enhancement, normalization, or region of interest segmentation for medical image data; missing feature engineering, meaning the study did not explain how features for model input were constructed, selected, or transformed from raw data in research involving prediction or classification modeling; or incomplete description of data sources, meaning the study claimed to conduct “multidimensional” or “multi-source” data analysis but actually used only a single dimension of data without reasonably explaining this limitation. To ensure consistency in the screening process, 2 reviewers (Ding T and Li S) independently evaluated studies based on the above criteria, and any study flagged by either as potentially noncompliant was reviewed by a third researcher (Wang Y) for final adjudication based on the specified points. The process for the specific selection of documents is shown in Figure 1.

To quantify the consistency of judgment between the 2 reviewers (Ding T and Li S) in applying the aforementioned inclusion and exclusion criteria, we calculated the Kappa statistic. For the initial independent screening results (inclusion/exclusion) of all 320 candidate documents, the 2 reviewers reached agreement on 304 documents (95.0%). The calculated Cohen’s Kappa coefficient was 0.83 (95% confidence interval: 0.75–0.91). According to the interpretation standards proposed by Landis and Koch^[12]^ (a Kappa value between 0.81 and 1.00 indicates “almost perfect” agreement), this result demonstrates excellent consistency between the reviewers in the literature screening process. For the 16 documents with discrepancies, all were reviewed and adjudicated by a third senior researcher (Wang Y) based on the established criteria, ensuring the final accuracy of the screening process.

### 2.3. Data analysis and network mapping

Bibliometrics serves as a vital tool for monitoring disciplinary development and knowledge dissemination patterns. This study employed CiteSpace (6.3.R1; developed by Chaomei Chen at Drexel University, Philadelphia) and VOSviewer (version 1.6.20; Centre for Science and Technology Studies, Leiden University, Leiden, Netherlands) to conduct an in-depth exploration of big data analysis in DKD. Through systematic extraction of core metadata, including journals, countries/regions, institutions, authors, references, and keywords, we constructed comprehensive knowledge network maps. The analysis focused on 3 principal dimensions: co-authorship network analysis examines multidimensional collaborative relationships among authors, institutions, and nations, effectively identifying productive research teams, leading institutional clusters, and international cooperation patterns^[13]^; co-citation analysis traces knowledge diffusion trajectories of pivotal literature, enabling researchers to grasp theoretical developments and core intellectual foundations^[14]^; and co-occurrence network analysis calculates semantic relationships among keywords, thereby accurately detecting research hotspots and frontiers within the field.^[14]^ All critical information was subsequently processed using Microsoft Office Excel 2007 (Microsoft Corporation) to create analytical charts and diagrams. The trend topics map was visualized using the Biblioshiny R package (R Core Team and distributed via CRAN [Comprehensive R Archive Network]).

### 2.4. Research ethics

The dataset utilized in this study was exclusively obtained from WoSCC, comprising solely publicly available academic records. As the data collection process was entirely based on published scholarly documentation without involving any collection of personal information from human participants, animal experimentation, or biological sample analysis, the use of these data raises no ethical concerns and requires neither ethical declaration nor approval procedures.

## 3. Results

### 3.1. Annual trend growth chart for publications

**Figure 2.**
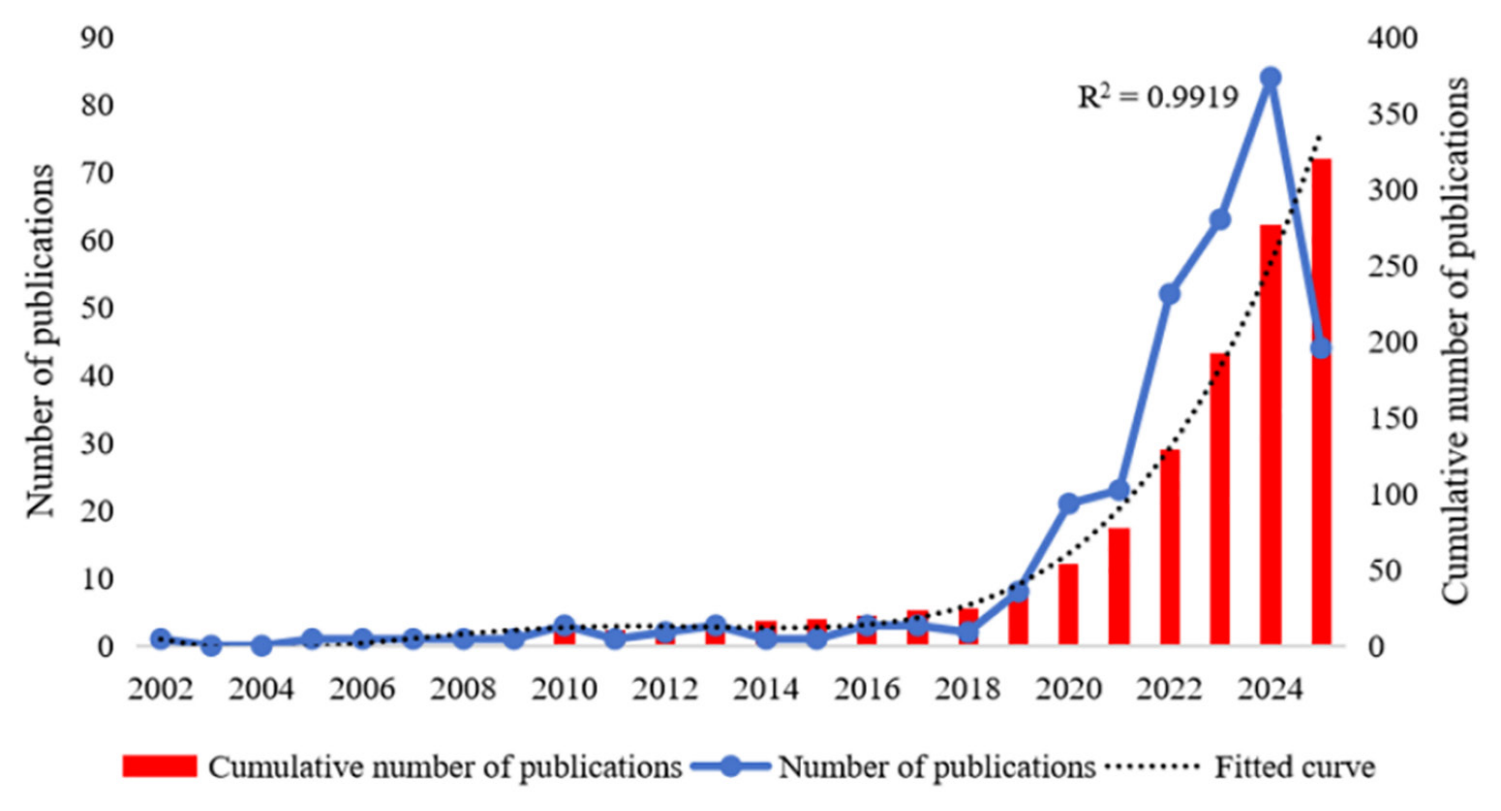
Annual publication trend chart.

The evolutionary trajectory of this research field can be mapped through temporal publication patterns. Our analysis identified 320 relevant publications (277 articles and 43 reviews) on big data analysis in the DKD field since the inception of WoS, representing contributions from 2176 researchers across 695 institutions in 51 countries. These studies collectively referenced 13,176 references from 3348 journals. As illustrated in Figure 2, both annual and cumulative publication outputs demonstrate a consistent upward trend. The earliest big data study on DKD emerged in 2002, followed by a period of limited activity (1.5 publications annually) through 2018. A marked acceleration in publication frequency became evident from 2019 onward, culminating in 84 publications in 2024. With 44 articles published in just the first 3 months of 2025, accounting for 52.4% of the total publications in 2024, and considering the overall upward trend in publications in this field since 2019, it is projected that big data research on DKD will maintain high research activity and publication growth in 2025.

### 3.2. Distribution of source journals and top 10 high-cited journals

**Figure 3.**
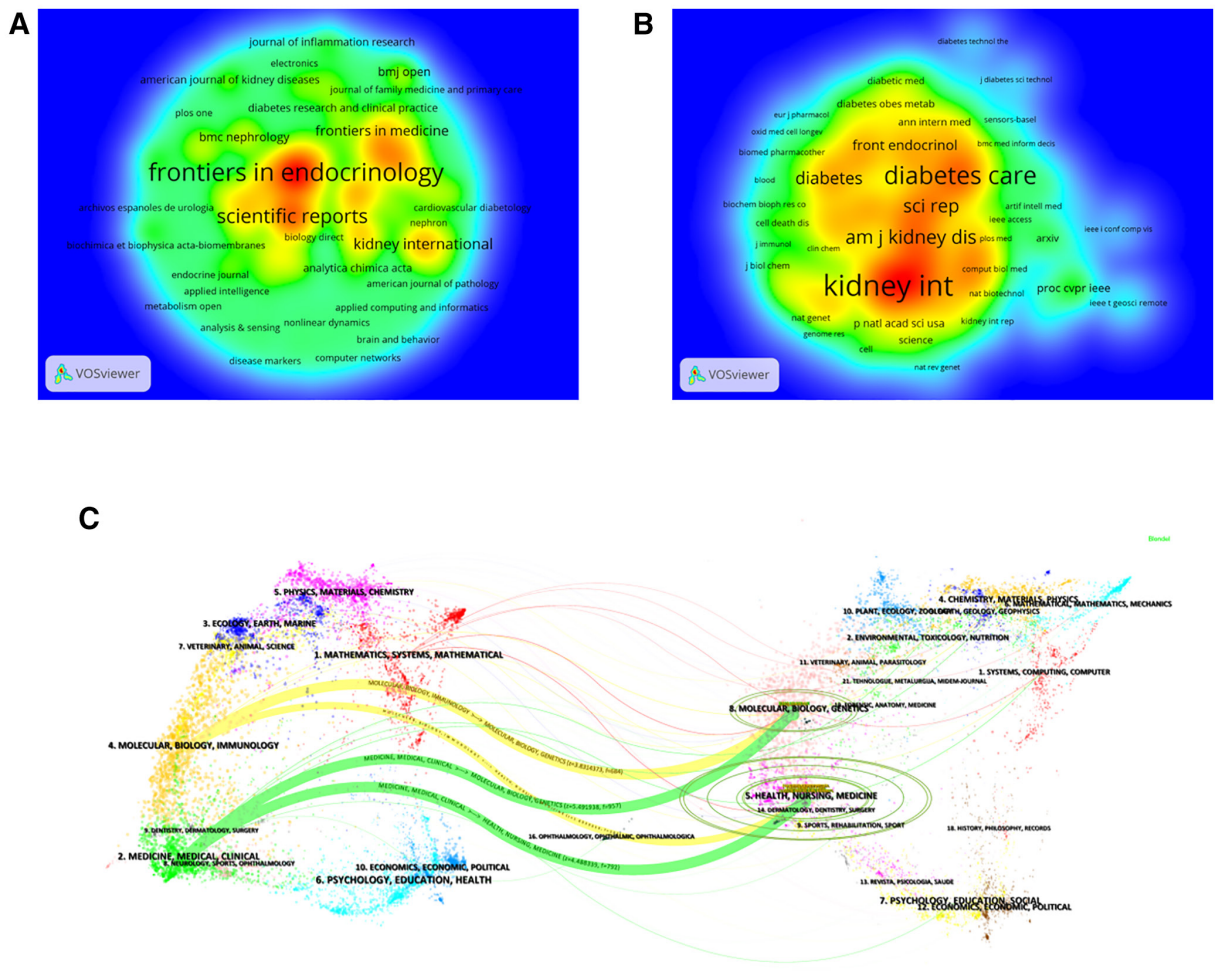
Journal analysis. (A) Citation coupling analysis of included journals, weighted by citations. (B) Co-citation analysis of the most frequently co-cited journals, weighted by citations. (C) The dual-map overlay of journals. Left: the citing journals; right: the cited journals.

The 320 retrieved papers were published across 192 different journals. The density plot (Fig. 3A) illustrated the distribution of publications among these journals. Table 1 specifically listed the top 10 journals in terms of publication volume for DKD big data analysis, accounting for 25.94% (83/320) of the total papers. The journal with the highest number of publications was *Frontiers in Endocrinology* (n = 21), followed by *Scientific Reports* (n = 13), with citation counts of 154 and 221, respectively. The Journal Citation Reports, an academic journal evaluation system developed by Thomson Reuters, categorizes global academic journals into 176 subject areas. Based on impact factor (IF) rankings, journals within each discipline are divided into 4 quartiles (Q1–Q4). Specifically, the top 25% of journals by IF are classified as Q1, those ranked 25% to 50% as Q2, those ranked 50% to 75% as Q3, and the remaining 25% as Q4. Among the top 10 journals, 7 were in Q1, 2 in Q2, and 1 in Q3. Notably, 2 journals with an IF exceeding 10 were *Kidney International* (166 citations) and the *Journal of the American Society of Nephrology* (421 citations), which also had the top 2 average citations per article (24 and 70, respectively) among all listed journals.

**Table 1:** The top 10 journals and co-cited journals related to big data research in diabetic kidney disease.

Rank	Sources	Count	Citations	Average citation	IF (2025)	JCR (2025)	Rank	Co-cited sources	Citations	IF (2025)	JCR (2025)
1	*Frontiers in Endocrinology*	21	154	7	3.9	Q2	1	*Kidney International*	455	14.8	Q1
2	*Scientific Reports*	13	221	17	3.8	Q1	2	*Journal of the American Society of Nephrology*	441	10.3	Q1
3	*Frontiers in Immunology*	7	73	10	5.7	Q1	3	*Diabetes Care*	355	14.8	Q1
4	*International Journal of Molecular Sciences*	7	76	11	4.9	Q1	4	*American Journal of Kidney Diseases*	249	9.4	Q1
5	*Kidney International*	7	166	24	14.8	Q1	5	*Scientific Reports*	230	3.8	Q1
6	*Medicine*	7	23	3	1.3	Q2	6	*Nephrology Dialysis Transplantation*	229	4.8	Q1
7	*Journal of the American Society of Nephrology*	6	421	70	10.3	Q1	7	*Nature Reviews Nephrology*	223	28.6	Q1
8	*Diabetes, Metabolic Syndrome and Obesity*	5	14	3	2.8	Q3	8	*Diabetes*	210	6.2	Q1
9	*Frontiers in Medicine*	5	16	3	3.1	Q1	9	*New England Journal of Medicine*	199	96.2	Q1
10	*Renal Failure*	5	67	13	3	Q1	10	*Diabetologia*	194	8.4	Q1

IF = impact factor, JCR = Journal Citation Reports, Q1 = quartile 1, Q2 = quartile 2, Q3 = quartile 3.

Co-citation analysis refers to the relationship formed when 2 publications are cited together by 1 or more subsequent articles. The higher the co-citation frequency, the stronger the academic relevance to the field. Figure 3B displays the density distribution of co-cited journals. Table 1 lists the top 10 most frequently co-cited journals, all of which belong to the Q1 category. Among them, 8 journals have co-citation counts exceeding 200. The most frequently co-cited journal is *Kidney International* (IF = 14.8), followed by *Journal of the American Society of Nephrology* (IF = 10.3) and *Diabetes Care* (IF = 14.8). A dual-map overlay effectively illustrates the disciplinary distribution and citation relationships between citing journals (left) and cited journals (right). As shown in Figure 3C, publications from journals in molecular biology, immunology, and clinical medicine predominantly cite literature from journals in health, nursing, biology, and genetics.

### 3.3. Distribution and co-authorship of countries/regions

**Figure 4.**
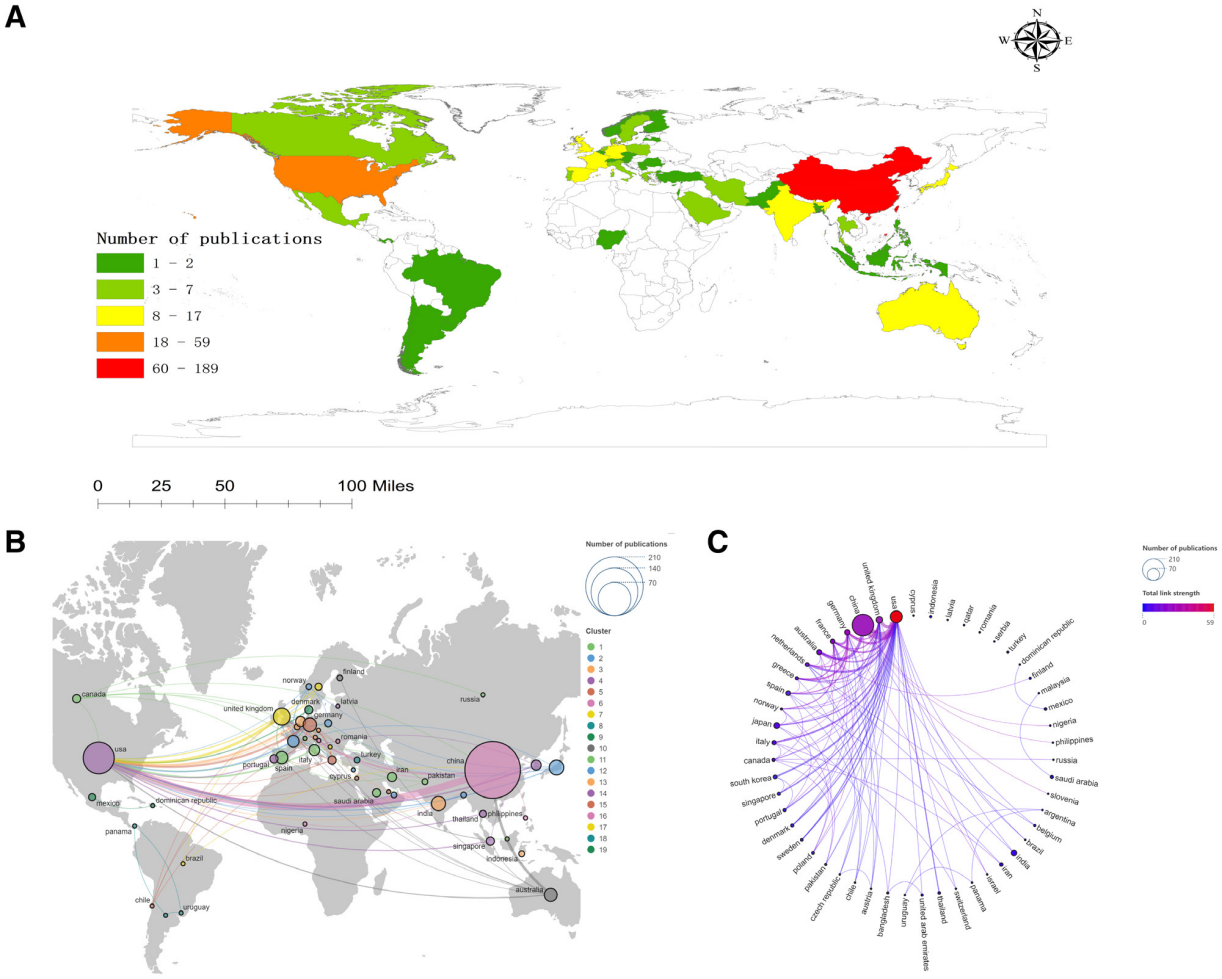
Collaboration distribution by countries/regions. (A) The world map of publications by countries/regions. (B) Specific inter-country cooperation. Bubble size represents publication volume by country/regions, while connection line thickness indicates collaboration intensity. (C) Chord diagram of country cooperation.

A total of 51 countries contributed to big data analysis in DKD, publishing 320 papers (Fig. 4A). Table 2 lists the top 10 most productive countries. China emerged as the most prolific contributor with 189 publications, accumulating 1449 citations. The United States of America (USA) ranked second in productivity (n = 59) but led in citation impact with 1885 citations. It is worth mentioning that Italy had the highest average citation count (56), followed by the USA (32), while China’s average citation count was only 8. The total link strength (TLS) metric reflects both the breadth (number of collaborating countries) and depth (frequency of collaboration) of a nation’s engagement in international research partnerships. Higher values indicate a more central role in global academic collaboration networks. The USA showed the highest TLS (n = 59), confirming its position as the central hub in international cooperation networks. Specific collaborative relationships between countries are illustrated in Figure 4B. The collaboration on this topic is mainly among China and the USA. To further elucidate the differences in research focus and citation impact between China and the United States, we summarized the relevant metrics in Supplementary Table 1, Supplemental Digital Content 1. Beyond the core China-USA cooperation, the chord diagram (Fig. 4C) also displays secondary cooperation characteristics. For example, the United Kingdom, Germany, and France form a close European cooperation network, while countries such as Japan and Australia serve as bridges connecting Asia and Western countries.

**Table 2:** The top 10 productive countries.

Rank	Country	Documents	Citations	Average number of citations	Total link strength
1	China	189	1449	8	27
2	USA	59	1885	32	59
3	United Kingdom	17	239	14	27
4	Japan	13	194	15	7
5	India	12	194	16	2
6	Germany	11	199	18	25
7	Australia	10	186	19	13
8	Spain	9	62	7	9
9	France	8	116	15	20
10	Italy	7	393	56	7

### 3.4. Distribution and co-authorship of institutions

**Figure 5.**
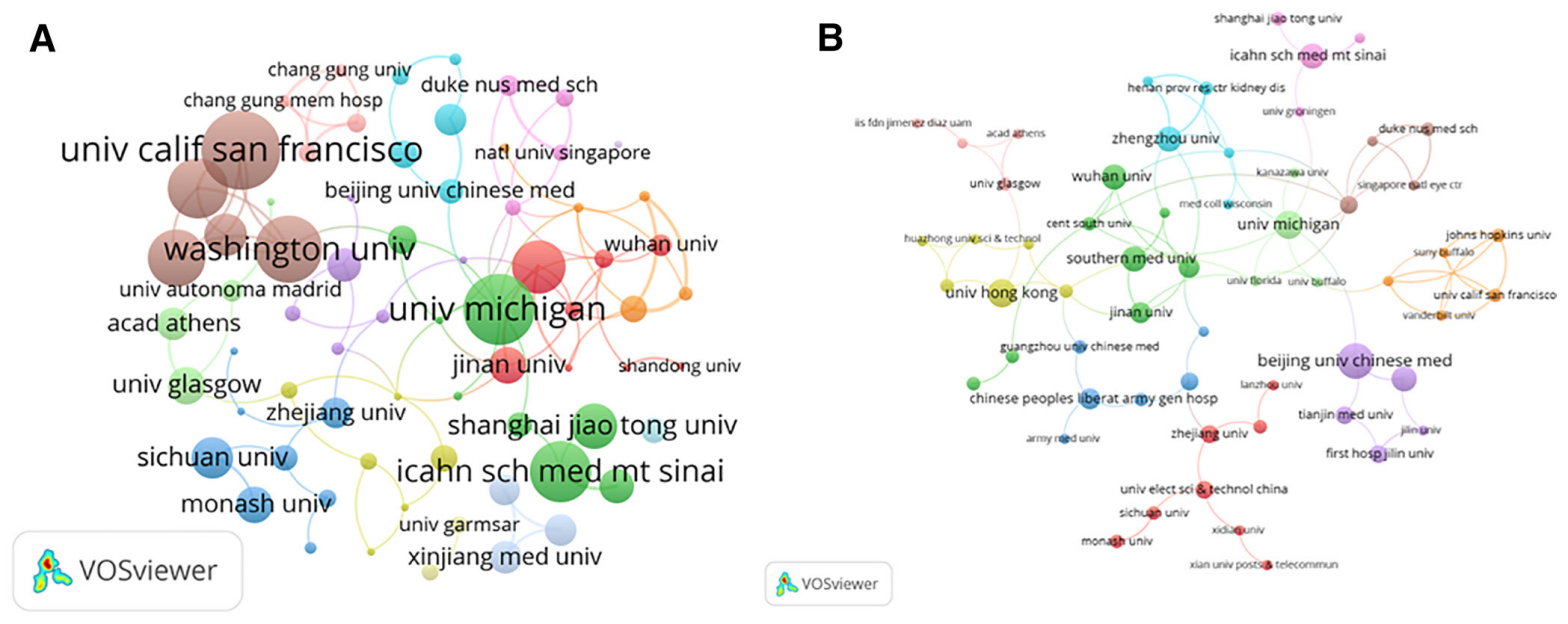
The co-authorship network of institutions. (A) The co-authorship map ranked by citation frequency. (B) The co-authorship map ranked by publication count.

A total of 695 institutions participated in DKD big data analysis. Table 3 lists the top 10 institutions ranked by citation count, comprising 8 USA institutions, 1 Italian institution, and 1 Swiss institution. We set a minimum publication threshold of 3 papers per institution, resulting in 70 qualifying institutions. The collaborative network among these 70 institutions was visualized using VOSviewer (Fig. 5). As shown in Figure 5A and Table 3, the University of California achieved the highest citation count (305 citations) from 4 publications, along with a TLS of 24. This was followed by the University of Michigan and Washington University, which published 8 and 3 papers, respectively, with citation counts and TLS of 258 and 38, and 235 and 16. Additionally, Beijing University of Chinese Medicine was the institution with the highest number of publications (10 publications), followed by the University of Michigan and the University of Hong Kong, both with 8 publications (see Fig. 5B and Supplementary Table 2, Supplemental Digital Content 2).

**Table 3:** The top 10 citations of institutions.

Rank	Institution	Country	Documents	Citations	Total link strength
1	University of California, San Francisco	USA	4	305	24
2	University of Michigan	USA	8	258	38
3	Washington University	USA	3	235	16
4	Icahn School of Medicine at Mount Sinai	USA	7	199	19
5	Fondazione IRCCS Ca’ Granda Ospedale Maggiore Policlinico	Italy	1	199	4
6	Geisel School of Medicine at Dartmouth	USA	1	199	4
7	Princeton University	USA	1	199	4
8	University of Zurich	Switzerland	1	199	4
9	Johns Hopkins University	USA	4	194	26
10	Medical University of South Carolina	USA	1	191	2

IRCCS = Istituto di Ricovero e Cura a Carattere Scientifico.

### 3.5. Distribution and co-authorship of authors

**Figure 6.**
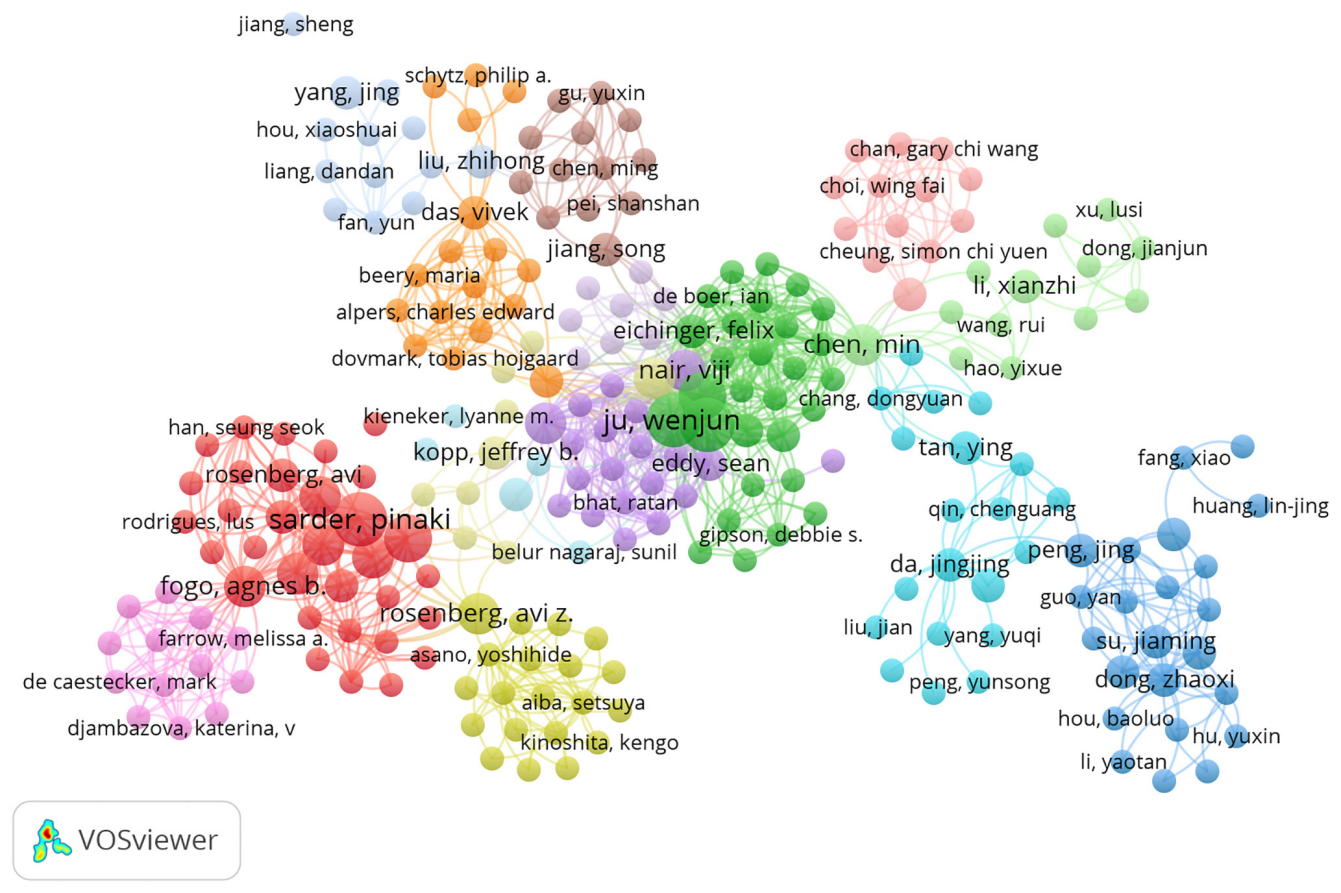
The co-authorship network of authors.

Analyzing authors in academic literature helps identify leading experts and key contributors within a research field. A total of 2176 researchers participated in big data studies related to DKD, collectively publishing 320 papers. Table 4 presents the top 10 most productive authors in this domain. Donovan, Michael J. and Coca, Steven G. from the Icahn School of Medicine at Mount Sinai each published 6 papers, with citation counts and average citations per paper of 100 and 17, and 94 and 16, respectively. Matthias Kretzler from the University of Michigan, while publishing 5 papers, achieved the highest citation metrics with 318 total citations and an impressive average of 64 citations per paper. Using VOSviewer, we conducted a co-authorship analysis of all 2176 scholars. After excluding authors without mutual connections, the remaining 248 authors formed a collaborative network divided into 15 distinct color-coded clusters (Fig. 6). The largest cluster, represented in red and centered around Sarder, Pinak, comprised 32 researchers. Supplementary Table 3, Supplemental Digital Content 3, specifically lists the top 10 authors by citation count, with Matthias Kretzler and Pinak Sarder ranking as the top 2.

**Table 4:** The top 10 productive authors.

Rank	Author	Organizations	Documents	Citations	Average citation	Total link strength
1	Donovan, Michael J.	Icahn School of Medicine at Mount Sinai	6	100	17	61
2	Coca, Steven G.	Icahn School of Medicine at Mount Sinai	6	94	16	60
3	Kretzler, Matthias	University of Michigan	5	318	64	73
4	Sarder, Pinaki	University of Florida	5	252	50	49
5	Ju, Wenjun	University of Michigan	5	251	50	72
6	Fleming, Fergus	Renalytix Incorporated	5	92	18	58
7	Cai, Guangyan	Chinese PLA General Hospital	5	69	14	87
8	Bitzer, Markus	University of Michigan	4	214	54	72
9	Tomaszewski, John E.	University at Buffalo	4	199	50	34
10	Nadkarni, Girish N.	Icahn School of Medicine at Mount Sinai	4	85	21	54

PLA = People’s Liberation Army.

### 3.6. Analysis of literature citations and reference co-citations

**Figure 7.**
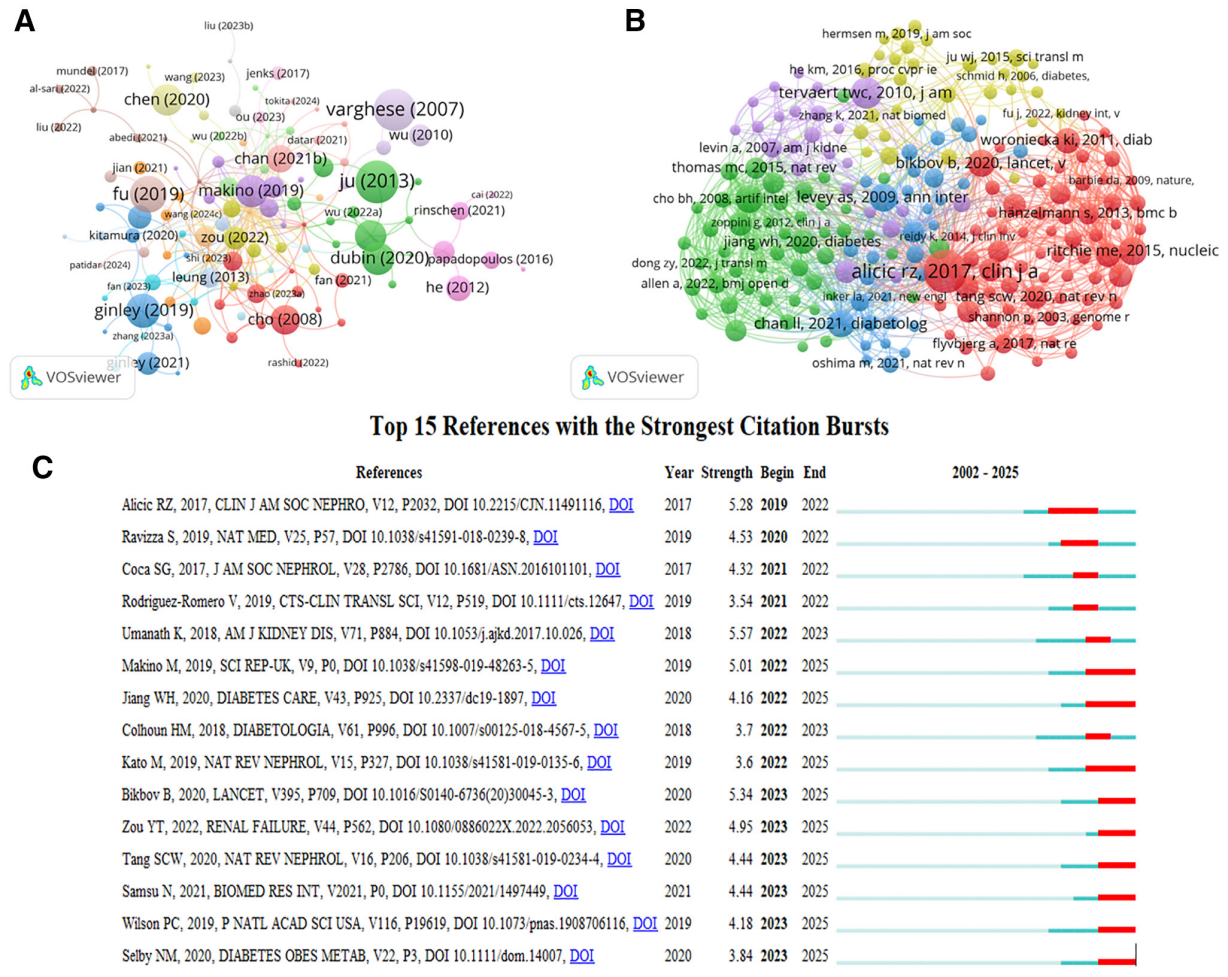
The analysis of included publications and their references. (A) Citation analysis of the included publications. (B) Co-citation analysis of the references. (C) Top 15 references with the strongest citation bursts by CiteSpace.

We conducted a citation analysis of the 320 included publications, with Table 5 listing the top 10 most-cited papers. The citation counts ranged from 92 to 199. Figure 7A presents the corresponding network visualization. The most frequently cited paper (199 citations) was published in *Genome Research* in 2013 under the title “Defining cell-type specificity at the transcriptional level in human disease.” The second most-cited paper in our analysis was published in the *Journal of the American Society of Nephrology* in 2007, titled “Urine biomarkers predict the cause of glomerular disease,” which has accumulated 191 citations to date. The third most-cited article (141 citations), published in *Molecular Metabolism* in 2019, was entitled “Diabetic kidney diseases revisited: a new perspective for a new era.”

**Table 5:** The top 10 highest-cited publications.

Rank	Title	Journals	First author	Type of essay	Citations	IF	PY
1	Defining cell-type specificity at the transcriptional level in human disease	*Genome Research*	Ju, Wenjun	Article	199	6.2	2013
2	Urine biomarkers predict the cause of glomerular disease	*Journal of the American Society of Nephrology*	Varghese, Sanju A	Article	191	10.3	2007
3	Diabetic kidney diseases revisited: a new perspective for a new era	*Molecular Metabolism*	Fu, Haiyan	Review	141	7	2019
4	Computational segmentation and classification of diabetic glomerulosclerosis	*Journal of the American Society of Nephrology*	Ginley, Brandon	Article	124	10.3	2019
5	Proteomics and metabolomics in kidney disease, including insights into etiology, treatment, and prevention	*Clinical Journal of the American Society of Nephrology*	Dubin, Ruth F	Review	111	8.5	2020
6	Artificial intelligence predicts the progression of diabetic kidney disease using big data machine learning	*Scientific Reports*	Makino, Masaki	Article	109	3.8	2019
7	Diabetic nephropathy and its risk factors in a society with a type 2 diabetes epidemic: a Saudi National Diabetes Registry-based study	*PLoS One*	Rubeaan, Khalid Al	Article	107	2.9	2014
8	Diabetic kidney disease: challenges, advances, and opportunities	*Kidney Diseases*	Chen, Ya	Review	105	3.2	2020
9	Integrated multi-omics approaches to improve classification of chronic kidney disease	*Nature Reviews Nephrology*	Eddy, Sean	Review	104	28.6	2020
10	Application of irregular and unbalanced data to predict diabetic nephropathy using visualization and feature selection methods	*Artificial Intelligence in Medicine*	Cho, Baek Hwan	Article	90	6.2	2008

IF = impact factor, PY = publication year.

We simultaneously conducted a co-citation analysis of the references cited in the included literature, with the corresponding network visualization presented in Figure 7B. A total of 13,176 references were incorporated into this analysis. Table 6 lists the top 10 most co-cited references. The most frequently co-cited reference was published in *Journal of the American Society of Nephrology*, titled “Diabetic kidney disease: challenges, progress, and possibilities” in 2017. Figure 7C displayed the top 15 references with the most significant citation bursts. The article by Umanath K (2018) demonstrated the strongest citation burst intensity at 5.57 occurrences from 2022 to 2023.

**Table 6:** The top 10 co-cited references.

Rank	Title	First author	Journals	Publication year	Citations
1	Diabetic kidney disease challenges, progress, and possibilities	Alicic, Radica Z	*Clinical Journal of the American Society of Nephrology*	2017	42
2	Pathologic classification of diabetic nephropathy	Tervaert, Thijs W	*Clinical Journal of the American Society of Nephrology*	2010	25
3	*limma* powers differential expression analyses for RNA-sequencing and microarray studies	Ritchie, Matthew E.	*Nucleic Acids Research*	2015	21
4	Artificial intelligence predicts the progression of diabetic kidney disease using big data machine learning	Makino, Masaki	*Scientific Reports*	2019	21
5	A new equation to estimate glomerular filtration rate	Levey, Andrew S	*Annals of Internal Medicine*	2009	21
6	Global, regional, and national burden of chronic kidney disease, 1990–2017: a systematic analysis for the Global Burden of Disease Study 2017	Bikbov, Boris	*Lancet*	2020	20
7	Derivation and validation of a machine learning risk score using biomarker and electronic patient data to predict progression of diabetic kidney disease	Chan, Lili	*Diabetologia*	2021	20
8	Transcriptome analysis of human diabetic kidney disease	Woroniecka, Karolina I	*Diabetes*	2011	18
9	clusterProfiler: an R package for comparing biological themes among gene clusters	Yu, Guangchuang	*Omics-A Journal of Integrative Biology*	2012	17
10	WGCNA: an R package for weighted correlation network analysis	Langfelder, Peter	*BMC Bioinformatics*	2008	16

BMC = BioMed Central, *limma* = linear models for microarray data, WGCNA = weighted gene co-expression network analysis.

### 3.7. The hotspots analysis of the keywords

**Figure 8.**
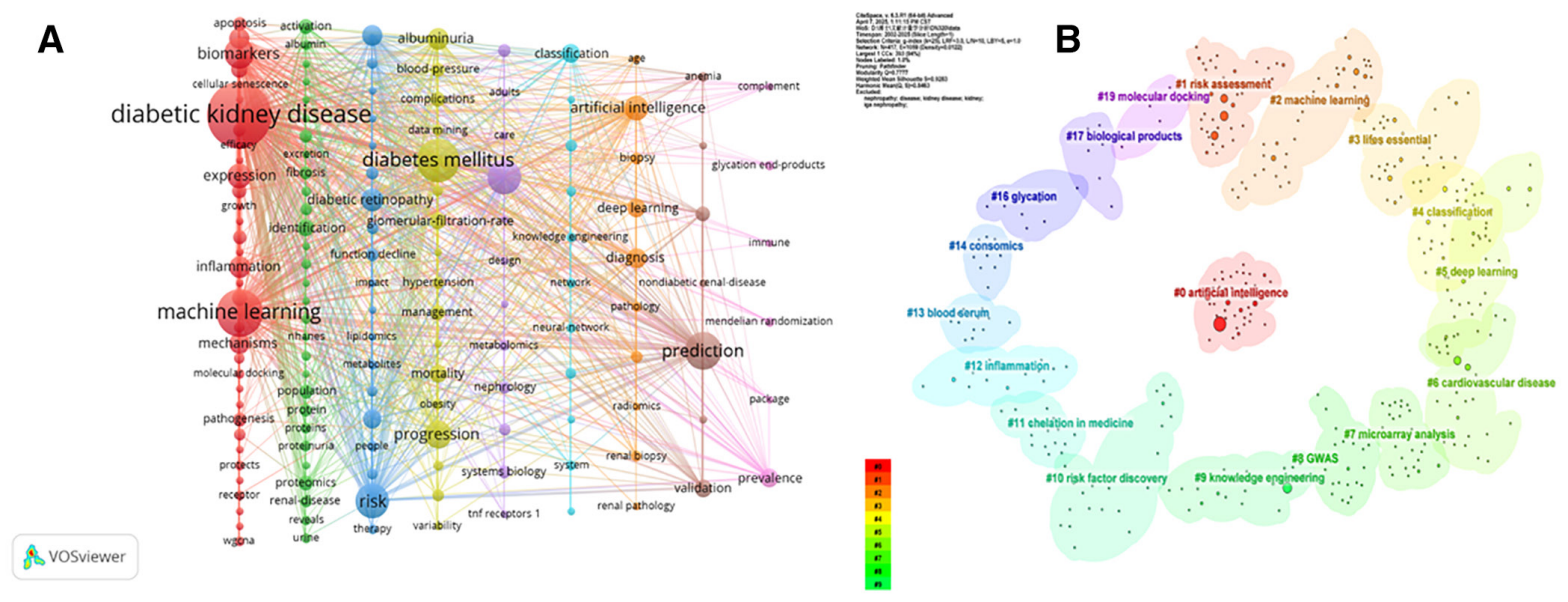
Analysis of keyword research hotspots. (A) Co-occurrence analysis of high-frequency keywords. (B) Cluster analysis of all keywords.

To identify research hotspots and directions in DKD big data analysis, we conducted a keyword co-occurrence analysis using VOSviewer on the 320 included publications. The initial extraction yielded 1527 keywords, which were refined to 1485 after merging synonyms and removing nonspecific terms. From this refined set, 146 high-frequency keywords were visualized (with a minimum occurrence of 3 times) in a co-occurrence network (Fig. 8A), where node labels represented keywords, node sizes indicated frequency, and connecting lines with varying thickness illustrated co-occurrence relationships and their strength. Table 7 presents the top 30 keywords by frequency, with the 10 most prominent being DKD (n = 192), ML (n = 99), diabetes mellitus (n = 82), prediction (n = 61), risk (n = 52), CKD (n = 51), biomarkers (n = 41), progression (n = 33), AI (n = 27), and expression (n = 27).

**Table 7:** The top 30 keywords.

Rank	Keywords	Counts	Total link strength
1	Diabetic Kidney Disease	192	520
2	Machine Learning	99	338
3	Diabetes Mellitus	82	283
4	Prediction	61	217
5	Risk	52	208
6	Chronic Kidney Disease	51	165
7	Biomarkers	41	137
8	Progression	33	142
9	Artificial Intelligence	27	84
10	Expression	27	71
11	Diabetic Retinopathy	22	82
12	Albuminuria	21	93
13	Inflammation	20	68
14	Bioinformatics	19	64
15	Association	18	82
16	Mechanisms	18	55
17	Diagnosis	17	69
18	Classification	16	53
19	Deep Learning	16	28
20	Prevalence	15	72
21	Outcome	15	54
22	Validation	13	66
23	Mortality	13	50
24	Glomerular Filtration Rate	12	59
25	Cells	12	50
26	Identification	12	22
27	Injury	11	35
28	Complications	10	53
29	Meta-Analysis	10	48
30	Gene	10	34

The clustering analysis of knowledge network mapping effectively reveals fundamental knowledge structures and core research hotspots in related fields. In this study, we employed CiteSpace software to conduct cluster analysis on the keywords (after merging synonyms and removing nonspecific terms), ultimately forming distinct clusters with unique characteristics. These clusters were clearly visualized through different color blocks in the mapping diagram (Fig. 8B). The clustering effectiveness was evaluated using 2 key metrics: Modularity *Q* and Mean Silhouette Coefficient *S*, with obtained values of 0.778 (>0.3) and 0.928 (>0.5), respectively, indicating a reasonable cluster structure with strong internal homogeneity. These keywords can be categorized into 18 cluster tags: AI (#0), risk assessment (#1), ML (#2), classification (#4), deep learning (DL, #5), knowledge engineering (#9), risk factor identification (#10), chelation therapy (#11), biological products (#17), molecular docking (#19), microarray analysis (#7), genome-wide association study (GWAS, #8), serum (#13), companion omics (#14), lifes essential’ (#3), CVD (#6), inflammation (#12), and glycosylation (#16).

### 3.8. The research trends and frontiers analysis of the keywords

**Figure 9.**
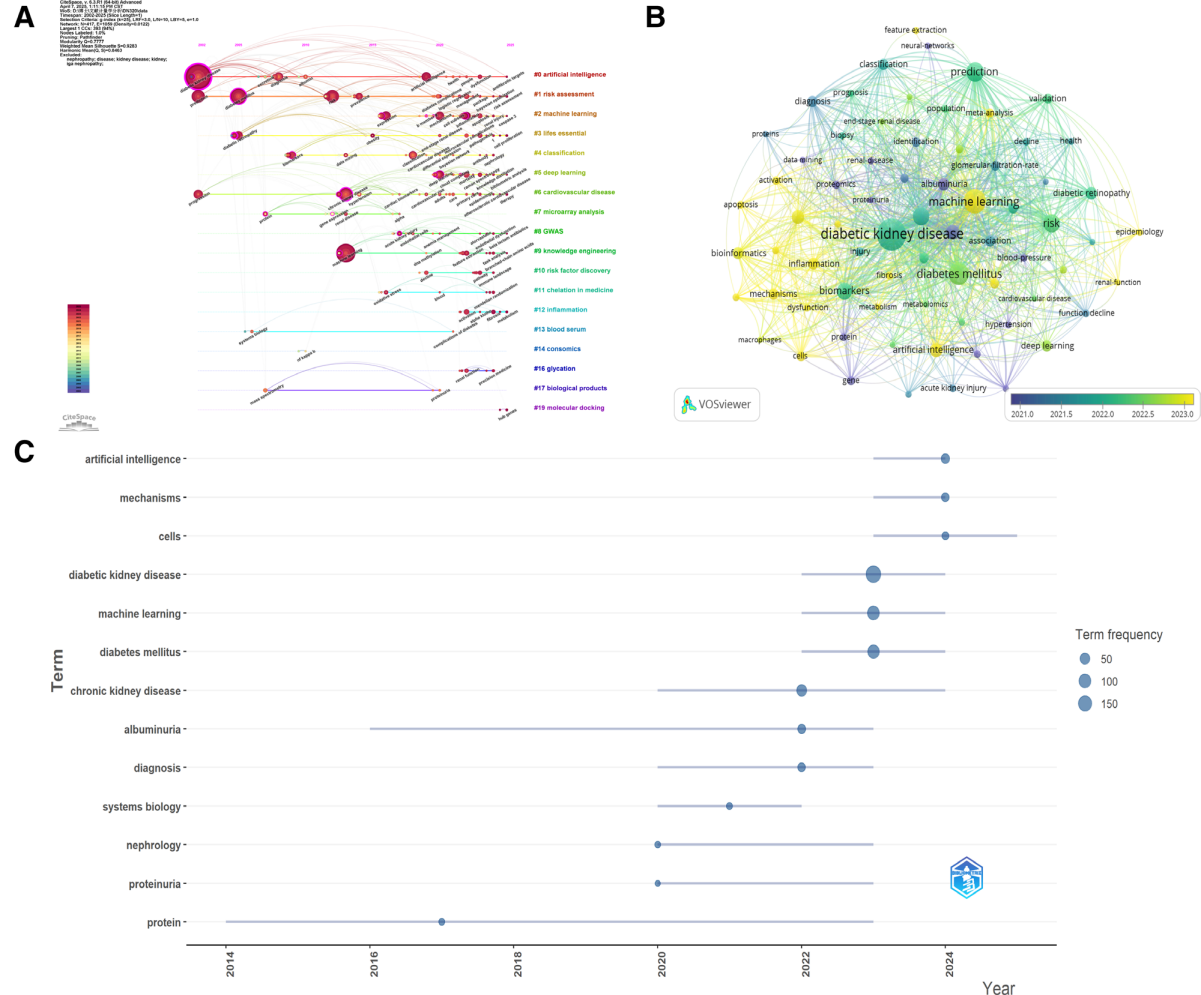
The analysis of the research frontiers of keywords. (A) Timeline chart of keywords. (B) Keyword overlay visualization analysis. (C) The trend topics map.

Through keyword co-occurrence analysis, this study further investigated emerging trends and frontiers in big data analysis for DKD research. CiteSpace was employed to generate a timeline visualization, which intuitively revealed the evolutionary trajectory of research themes. As shown in Figure 9A, from 2002 to the present, both the research scope and scale have progressively expanded, with increasingly strengthened connections. The keyword overlay visualization analysis (Fig. 9B) revealed that ML, AI, bioinformatics, mechanisms, cells, and inflammation have gained prominence in recent years. Similarly, the trend topics map (Fig. 9C) indicated that current research frontiers in big data-driven DKD studies primarily focus on ML, AI, mechanisms, and cells.

## 4. Discussion

Compared with traditional literature reviews, bibliometric analysis enables a systematic and comprehensive evaluation of published works in a specific research domain. This study employed bibliometrics to synthesize collaborative networks, research hotspots, and future directions in big data analysis for DKD research. The findings suggested that publication output has evolved from gradual growth to rapid expansion. Although our data collection only extended through March 2025, the publication count in 2025 (44 publications) has already surpassed half of 2024’s total output (84 publications). Although the data for 2025 are incomplete and preclude rigorous year-on-year comparisons, the recording of 44 articles as of March 2025 indicates sustained high publication activity. Combined with the overall rapid growth trend since 2019, it is highly likely that the field will continue its growth momentum in 2025 and beyond. This preliminary observation requires validation with complete annual data in the future.

Several factors may account for the rapid increase in big data-related DKD publications after 2019. First, the widespread adoption of AI and ML in biomedical research during this period provided powerful analytical tools for handling high-dimensional clinical and omics data. Second, the exponential growth of multi-omics technologies (e.g., transcriptomics, proteomics, and metabolomics) enabled comprehensive molecular profiling of DKD, generating large-scale datasets suitable for big data analysis. Third, the global emphasis on precision medicine, particularly for complex diseases such as DKD, encouraged the development of predictive models and biomarker discovery using large patient cohorts. Fourth, the establishment of large public databases (e.g., GEO, TCGA, and UK Biobank) and EHR systems facilitated data sharing and reuse, lowering the barrier for big data research. Collectively, these technological and infrastructural advancements have created a favorable environment that has accelerated research output in this field since 2019.

This study included 320 publications comprising original articles and reviews, contributed by 2176 researchers from 695 institutions across 51 countries, indicating that big data analysis in DKD has garnered widespread global attention. Among the top 10 most productive journals, 9 were classified as Q1 or Q2. *Frontiers in Endocrinology* (21 publications) ranked highest in publication output. Co-citation analysis of journals showed that all top 10 cited journals belonged to Q1, with *Kidney International* being the most frequently cited (455 citations). These findings demonstrate that the field currently exhibits both substantial research scale and high-quality output.

The USA served as the central hub for transnational collaboration. Although its publication output was only one-third of China’s (59 publications), it ranked first in both citation count and TLS and established close collaborations with multiple countries, including China, Germany, the United Kingdom, France, and Denmark. Furthermore, among the top 10 institutions by citation count, 8 were from the USA. The high citation counts and TLS values suggested a strong influence from American institutions. Although China demonstrated the highest productivity (189 publications), its total citation count (1449), average citations per paper (8), and TLS (27) lagged significantly behind the USA. This indicated a persistent gap in high-impact research and global influence from Chinese institutions. Similar regional disparities observed in other countries underscore the urgent need for enhanced international collaboration and knowledge exchange.

This disparity in citation impact may be attributed to fundamental differences in research focus and innovation capacity between the 2 countries. Publications from the USA predominantly concentrate on pioneering mechanistic discoveries, novel biomarker identification, and the development of original AI/ML models. These studies, often published in high-impact nephrology and computational biology journals, tend to establish foundational knowledge and methodological benchmarks, thereby attracting widespread international citations.^[15,16]^ In contrast, a considerable proportion of research from China appears more application-oriented, focusing on clinical validation of existing models, epidemiological investigations, and network pharmacology analyses of traditional herbal medicine.^[17,18]^ While such research holds significant clinical relevance for regional healthcare, its novelty and global generalizability may be comparatively limited, leading to slower accrual of international citations. This pattern suggests that bridging the influence gap requires not only increased international collaboration but also a strategic shift towards more exploratory, mechanism-driven research that addresses globally recognized challenges in DKD pathogenesis and precision medicine.

The co-authorship analysis indicates that Donovan, Michael J.^[16,19]^ and Coca, Steven G.^[19]^ from the USA demonstrate the highest research productivity. Their work primarily focuses on advancing precision risk stratification and personalized therapies through the development of biomarkers for kidney diseases. Among the top 10 productive authors, Matthias Kretzler and Pinaki Sarder ranked in the top 2 in citation counts. Matthias Kretzler is a professor of Internal Medicine, Computational Medicine, and Bioinformatics at the University of Michigan. He specializes in DKD and nephrology research. His team’s publications concentrate on precision diagnosis and treatment of DKD, integrating multi-omics data with computational models to elucidate disease mechanisms and develop predictive models, thereby advancing molecular classification and targeted interventions for kidney diseases.^[20]^ Meanwhile, Pinaki Sarder is a professor at the University of Florida, located at the central hub of the largest collaboration cluster, who leads research focused on integrating multimodal kidney data and developing renal pathological image analysis tools to facilitate clinical translation.^[21]^

Highly cited publications typically represent seminal findings within a given field, where original research articles and review papers often reflect the most recent advances in relevant areas. This study analyzed 320 publications related to big data analysis for DKD research, from which the top 10 cited research papers were selected for evaluation. New researchers can refer to these publications to quickly become familiar with the research progress in this field. In 2013, Ju et al published a seminal article titled “Defining cell-type specificity at the transcriptional level in human disease” in *Genome Research*.^[22]^ Cell lineage-specific transcripts are critical for the function of differentiated tissues and may mediate acquired chronic diseases such as diabetes. However, genome-wide experimental identification of cell lineage-specific genes remains technically challenging in most human solid tissues. This study developed a novel ML-based genome-scale method that computationally identified cell lineage-specific genes for the first time, using kidney podocytes as a validation model (achieving 65% accuracy, significantly surpassing the 23% accuracy of experimental mouse models). The approach successfully detected genes associated with hereditary and CKDs (including DKD), whose expression levels strongly correlated with patients’ renal function decline. This research provides a universal computational tool adaptable to other tissues or diseases, establishing a foundation for developing organ-specific therapeutic strategies.

*Journal of the American Society of Nephrology* published an article titled “Urine biomarkers predict the cause of glomerular disease” by Varghese et al in 2007.^[23]^ This study successfully established a noninvasive diagnostic model by combining urine proteomics with artificial neural networks, which could differentiate glomerular diseases including DKD (sensitivity 75%–86%, specificity 67%–92%). Further research identified 11 charge isoforms of plasma proteins as key biomarkers for disease discrimination. This approach may provide potential targets for developing clinical detection methods to replace renal biopsy. The paper “Diabetic kidney diseases revisited: a new perspective for a new era” was published by Fu et al in 2019 in *Molecular Metabolism*.^[24]^ This comprehensive review summarized alterations in the diabetic kidney microenvironment, genetic/epigenetic mechanisms, epidemiological changes, and clinical management challenges, while exploring the potential of integrating systems biology with translational medicine in current healthcare systems during the big data era. These research advancements are expected to provide novel insights for addressing existing clinical challenges in DKD. Subsequently, in 2020, Chen et al further clarified the diverse clinical manifestations and treatment difficulties of DKD.^[25]^ While acknowledging therapeutic advances, including novel inhibitors and the identification of some predictive biomarkers, they emphasized the ongoing need to develop more precise diagnostic and predictive tools to improve clinical management outcomes.

In 2019, Ginley et al published a study titled “Computational segmentation and classification of diabetic glomerulosclerosis” in *Journal of the American Society of Nephrology*.^[26]^ The study developed a digital workflow integrating conventional image analysis with DL for the automated classification of DKD renal biopsies. This approach simplified complex glomerular structures into 3 components and achieved high-precision detection (>93% accuracy in key structure identification) using convolutional neural networks and unsupervised techniques, with classification results comparable to pathologists’ interpretations (κ = 0.48–0.68). This technology shows potential to reduce interobserver variability and provide more objective quantitative support for clinical decision-making. Building upon this paradigm of computational renal pathology, a recent study has further extended DL-based classification to renal cell carcinoma using whole-slide images, achieving robust performance through a transformer-based multi-instance learning architecture, which underscores the broader applicability of AI-driven histopathological analysis in nephrology.^[27]^

Dubin et al in 2020 published a review on omics applications in kidney disease in *Clinical Journal of the American Society of Nephrology*.^[28]^ The authors concluded that advances in mass spectrometry and high-throughput detection technologies combined with ML analysis have facilitated breakthroughs, including the identification of membranous nephropathy antigens and the discovery of inflammatory mediators in DKD. Future research should focus on enhancing multi-omics integration, longitudinal validation, and kidney tissue-specific investigations. In the same year, Eddy et al expressed similar views in *Nature Reviews Nephrology*, proposing that multi-omics technologies (genomics, proteomics, and metabolomics) combined with ML could reveal molecular-level disease mechanism differences in CKD, thereby advancing precision classification and personalized treatment approaches.^[29]^

In 2019, Makino et al published a study titled “Artificial intelligence predicts the progression of diabetic kidney disease using big data machine learning” in *Scientific Reports*.^[30]^ By applying natural language processing and longitudinal big data ML to EHRs from 64,000 diabetic patients, the study developed a predictive model for DKD progression with 71% accuracy, capable of identifying high-risk patients 6 months in advance, thereby providing a basis for early intervention to reduce ESRD. *PLoS One* published an epidemiological investigation on DKD by Al-Rubeaan et al in 2014. The study, based on data from 54,670 type 2 diabetes mellitus patients in Saudi Arabia, revealed a high DKD prevalence rate of 10.8%, with major risk factors including long disease duration, advanced age, and comorbidities, highlighting the urgent need for targeted screening and prevention programs in the country.^[31]^

“Application of irregular and unbalanced data to predict diabetic nephropathy using visualization and feature selection methods” was published by Cho et al in *Artificial Intelligence in Medicine* in 2008.^[32]^ This research demonstrated that ML methods, including support vector machines and feature selection techniques, could accurately predict DKD onset from irregular diabetes data, achieving significantly improved performance compared with conventional approaches. Additionally, the team developed a visualization tool to assist physicians in analyzing risk factors and optimizing treatment decisions.

Co-cited references refer to 2 or more publications that are cited together by other works. This study lists the top 10 frequently co-cited references, which align with the aforementioned research themes and primarily focus on: Disease-focused studies: clarifying pathological classifications of DKD, addressing clinical challenges and global disease burden, and formulating corresponding management strategies^[33]^; Mechanistic investigations: deciphering molecular mechanisms of DKD through omics technologies and gene network analysis^[34]^; and Clinical risk prediction: developing AI models for DKD based on clinical data, biomarkers, and bioinformatics tools.^[15,30,35]^ Future research is expected to further leverage model optimization and multi-omics integration to explore disease etiology and improve patient diagnosis and treatment.

**Figure 10.**
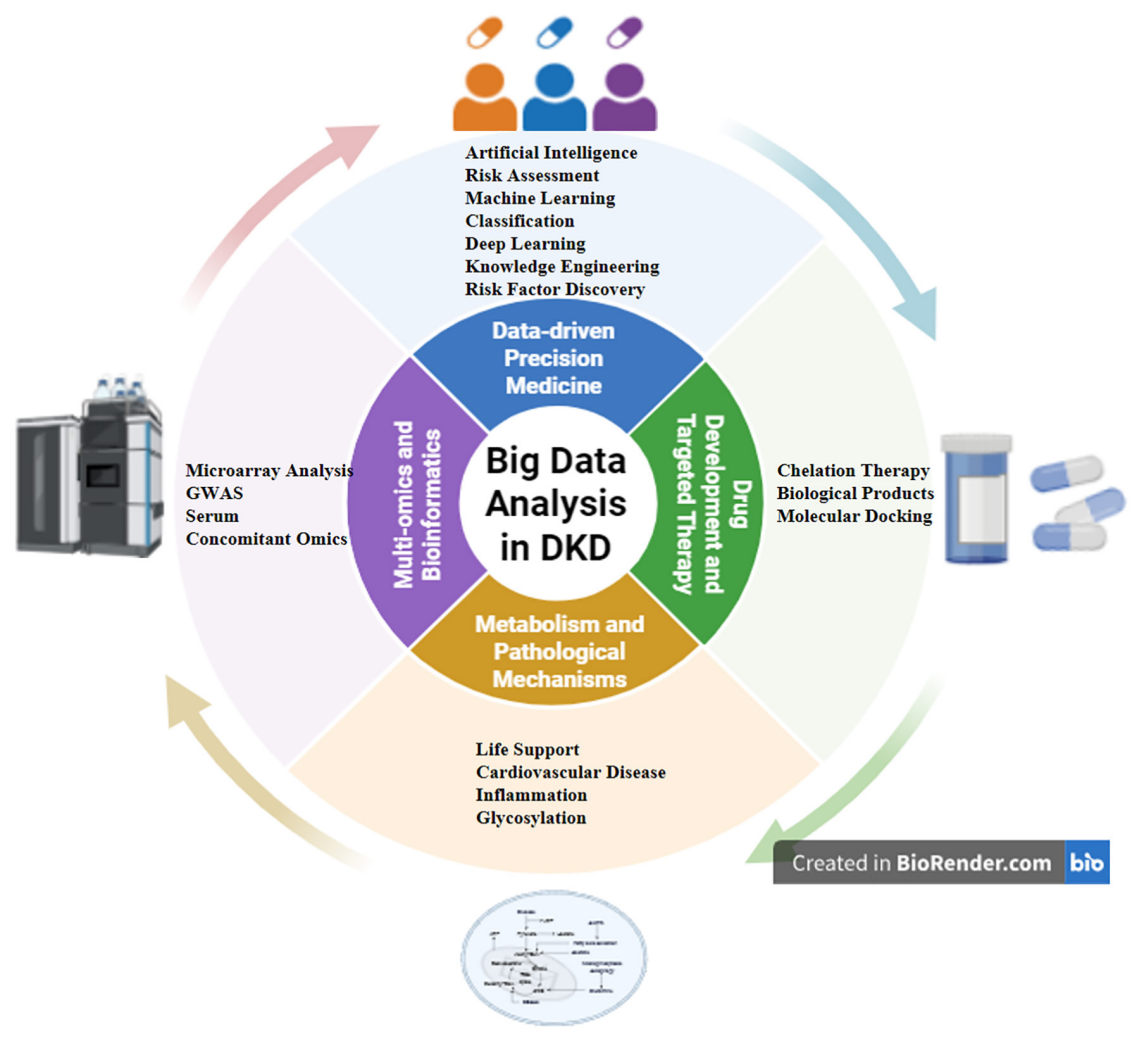
Four major research perspectives in DKD big data analysis. DKD = diabetic kidney disease, GWAS = genome-wide association study.

High-frequency keywords can reflect research hotspots and directions in a field. The most representative high-frequency keywords include DKD, ML, diabetes mellitus, prediction, risk, CKD, biomarkers, progression, AI, and expression. These keywords clearly indicate that research on big data analysis in DKD primarily focuses on using AI models for risk prediction and progression assessment of CKD in diabetic patients. Additionally, this study conducted a cluster analysis of all keywords to visually present the research scope of this field. The 18 clusters revealed 4 major research perspectives in DKD big data analysis over the past 2 decades: Data-driven precision medicine: AI, risk assessment, ML, classification, DL, knowledge engineering, and risk factor discovery; Drug development and targeted therapy: chelation therapy, biologics, and molecular docking; Multi-omics and bioinformatics: microarray analysis, GWAS, serum, and companion omics; and Metabolic and pathological mechanisms: life support, CVD, inflammation, and glycosylation (see Fig. 10).

To delineate research frontiers, this study employed the keyword timeline chart, the overlay visualization map, and the trend topics map. The findings demonstrate that big data analysis in DKD has progressively expanded in scale over 2 decades, with the continuous emergence of new themes. Current research frontiers are identified as ML, AI, mechanisms, and cells, which align with conclusions emphasized across multiple recent publications.^[18,36]^ AI is a multidisciplinary field of computer science that has rapidly advanced over the past decade due to enhanced computational power and data proliferation.^[37]^ It aims to endow machines (e.g., computers or robots) with capabilities to simulate, extend, and implement human intelligence, including learning, reasoning, decision-making, perception, and language processing.^[38]^ ML, as a core subfield of AI, serves as the key technological pillar for enabling intelligent decision-making in AI systems.^[39]^ Currently, DKD screening primarily relies on microalbuminuria and glomerular filtration rate measurements.^[40]^ However, these conventional biomarkers face limitations such as nonspecificity, diagnostic lag, and technical constraints,^[41]^ making them inadequate for meeting the growing demands of early diagnosis and precision medicine. AI models, particularly those based on ML, have emerged as pivotal tools for early and precise DKD management.^[42]^ ML exhibits exceptional data-driven capabilities, excelling in processing multi-source heterogeneous data (encompassing genomics, metabolomics, lipidomics, imaging data, and clinical indicators).^[42]^ Through feature dimensionality reduction, it extracts critical biomarkers and enables real-time dynamic analysis.^[42]^ Current ML algorithms applied to DKD research mainly include supervised learning (logistic regression, random forest, support vector machine, artificial neural network, K-nearest neighbor, eXtreme gradient boosting, and decision tree),^[43]^ unsupervised learning (two-step clustering, K-means, hierarchical clustering, and principal component analysis),^[44]^ and DL (convolutional neural networks, recurrent neural networks, and transformers).^[45]^ These AI-powered modeling approaches have significantly improved DKD prediction accuracy and advanced precision medicine growth. Beyond DKD, the utility of AI in renal imaging-based diagnosis is also gaining momentum. For instance, an AI model built on non-contrast CT has recently demonstrated high diagnostic accuracy for clear-cell renal cell carcinoma, highlighting the potential of noninvasive imaging combined with ML for renal disease detection, a strategy that may also inform future DKD imaging research.^[46]^

Beyond ML-based modeling approaches, other big data analysis technologies (particularly high-throughput techniques) have been applied to DKD for multidimensional molecular mechanism exploration. Microarray analysis, a high-throughput gene expression profiling technology based on DNA chips, enables genome-wide molecular characterization through hybridization between solid-phase probes and RNA/cDNA samples.^[47]^ Numerous studies have utilized kidney tissue, urine, and serum microarray datasets from the GEO database to elucidate DKD pathogenesis,^[48]^ screen potential biomarkers,^[49]^ and identify novel therapeutic targets.^[50]^ GWAS systematically identify genetic variants associated with DKD susceptibility,^[51]^ providing foundations for risk prediction, such as the reported rs56094641 locus in *FTO*^[52]^ and rs3765156 in *PIK3C2B*.^[53]^ Importantly, genetic polymorphisms in inflammation-related genes have also gained attention as potential DKD biomarkers: a comprehensive meta-analysis of Asian populations found that the interleukin-6 gene rs1800795 (G>C) variant is closely associated with T2D susceptibility (a major risk factor for DKD) and inflammatory responses, highlighting its potential as a genetic biomarker for DKD risk stratification.^[54]^ Companion omics integrate multi-omics data to systematically reveal interactions among DKD-related biomolecules. For example, an integrated urine metabolomics-peptidomics analysis uncovered 16 progressively regulated biomarkers (10 metabolites and 6 peptides) across DKD stages, implicating amino acid metabolism and cellular protein processing as core pathological mechanisms.^[55]^ Collectively, these bioinformatics technologies provide transformative perspectives for deciphering DKD mechanisms.

The treatment of DKD has always been a focus in the medical field. Currently, some breakthrough therapeutic approaches based on big data analysis, such as chelation therapy, biological products, and molecular docking, can promote precision treatment of DKD from different perspectives and provide strong technical support for targeted drug development. For example, the application of ML and bioinformatics can effectively assist in the precise implementation of chelation therapy for DKD, including identifying subgroups with abnormal metal metabolism and optimizing chelator selection and dosage regimens.^[56]^ Additionally, some biological products for treating DKD that have been approved or are in preclinical/clinical trials, including anti-inflammatory monoclonal antibodies,^[57]^ cytokines,^[58]^ and RNA-targeting agents,^[59]^ have been developed based on the analysis of various large biological databases. Meanwhile, molecular docking technology can match active drug components with molecules in DKD target databases to construct protein-protein interaction networks and identify core targets.^[17]^ Notably, network pharmacology combined with molecular docking has also been successfully applied to explore the mechanism of traditional Chinese medicine in treating metabolic diseases related to DKD: a recent study confirmed that *Zanthoxylum bungeanum*, a TCM with edible and medicinal value, exerts antidiabetic effects by targeting AKT serine/threonine kinase 1, interleukin-6, and other key molecules through its active components (e.g., diosmetin and quercetin), providing new ideas for developing DKD-targeted drugs from natural products.^[60]^

This study employed bibliometric methods to comprehensively analyze the evolving trends and research hotspots in the integration of big data analysis into DKD research; yet, several limitations should be acknowledged. First, although WoSCC is widely recognized as one of the most authoritative and standardized databases for bibliometric analysis, and its stringent inclusion criteria help ensure data quality and consistency, relying on a single database may limit the comprehensiveness of literature coverage to some extent. For instance, some regional journals or specialized core literature not indexed in WoSCC (e.g., studies included in databases such as Scopus, PubMed, and Embase) may have been omitted, which could affect the representativeness of the sample in specific geographical regions (such as East Asia) or specialized research directions. In particular, regionally focused studies published in journals indexed primarily in local or specialty databases (e.g., some PubMed-indexed journals with a regional focus) might not be captured, potentially influencing the generalizability of findings to all research contexts. Future studies may consider integrating multiple authoritative databases for cross-retrieval and data fusion to more comprehensively depict the global knowledge landscape of the field and further validate the robustness of the conclusions drawn in this study. Second, high citation counts do not necessarily reflect superior quality, as frequent citations may stem from a study’s controversial nature rather than its academic merit, and self-citations or reciprocal citations among authors could artificially inflate citation metrics. Third, the inherent time lag between publication and citation may lead to an underestimation of emerging research areas in bibliometric analysis. Fourth, as our analysis primarily relied on textual information such as titles, abstracts, and keywords, it may not fully capture the nuanced conceptual depth or core contributions of individual studies. Fifth, while our analysis highlighted quantitative disparities in research output and influence between major contributing countries such as the USA and China, it did not employ text-mining techniques (e.g., topic modeling or natural language processing on abstracts) to qualitatively dissect the differences in research themes, methodological innovation, or clinical translation focus. Future bibliometric studies could integrate such content analysis to provide a more nuanced understanding of the drivers behind national research profiles and their impact. Finally, the publication data for 2025 included in this study covered only the first 3 months (up to March 26, 2025). Therefore, any comparison with full-year data from previous years and projections about the annual trend for 2025 should be interpreted with caution.

## 5. Conclusion

In summary, this study employed bibliometric analysis to systematically characterize the research trends, collaboration patterns, and frontier hotspots in big data analysis for DKD. Research in this area showed an upward trajectory, with extensive collaborations established among multiple countries, institutions, and authors. Currently, applying AI models (primarily ML-based) for risk prediction and progression assessment of CKD in diabetic patients represents a key research focus, and multi-omics technologies have achieved important breakthroughs in the screening and identification of DKD diagnostic/prognostic biomarkers, which form a high-dimensional biological data foundation for the optimization and improvement of AI/ML models; in turn, AI/ML models effectively excavate the potential clinical value and molecular regulatory relationships of multi-omics biomarkers, realizing the deep integration of biological discovery and data-driven prediction. Meanwhile, AI, ML, mechanisms, and cells constitute the research frontiers of this field. These findings may provide valuable references for scholars dedicated to investigating DKD mechanisms and developing precision diagnostics and therapeutics.

## Acknowledgments

The authors are grateful to all participants for their contributions to this study.

## Author contributions

**Conceptualization:** Mingkang Zhang, Yazhi Wang.

**Data curation:** Tingting Ding, Shang Li, Mingkang Zhang, Yazhi Wang.

**Methodology:** Tingting Ding.

**Investigation:** Qinglin Guo.






