# Supplementary material for: Bibliometrics and visualization analysis: Big data research on diabetic kidney disease from 2000 to 2025
Source: Medicine (Baltimore). 2026 Aug 14;105(33):e50234. doi: 10.1097/MD.0000000000050234 (PMC13480767; doi:10.1097/MD.0000000000050234)
Supplement: Supplementary file 1 [file medi-105-e50234-s001.docx]

**Supplementary Table 1.** Comparison of research focus and citation impact between China and the United States

| Research Sub-direction | Country | Number of publications (Proportion) | Average citations per publication |
| --- | --- | --- | --- |
| Mechanism Research (e.g., multi-omics, pathways) | China | 30 (15.9%) | 8.1 |
|  | USA | 6 (10.2%) | 104.5 |
| Biomarker Screening (e.g., diagnostic/prognostic) | China | 56 (29.6%) | 15.6 |
|  | USA | 13 (22.0%) | 52.8 |
| Traditional Chinese Medicine (TCM) (e.g., network pharmacology) | China | 8 (4.2%) | 11.9 |
|  | USA | 0 (0.0%) | 0 |
| Clinical prediction models and epidemiology | China | 82 (43.4%) | 12.2 |
|  | USA | 21 (35.6%) | 36.4 |

Abbreviations:TCM=Traditional Chinese Medicine, e.g.=exempli gratia. USA=United States of America
