# Supplementary material for: Bibliometrics and visualization analysis: Big data research on diabetic kidney disease from 2000 to 2025
Source: Medicine (Baltimore). 2026 Aug 14;105(33):e50234. doi: 10.1097/MD.0000000000050234 (PMC13480767; doi:10.1097/MD.0000000000050234)
Supplement: Supplementary file 2 [file medi-105-e50234-s002.docx]

**Supplementary Table 2.** The top 10 productive institutions.

| Rank | Institution | Country | Documents | Citations | Average number of citations | Total link strength |
| --- | --- | --- | --- | --- | --- | --- |
| 1 | Beijing University of Chinese Medicine | China | 10 | 45 | 5 | 4 |
| 2 | University of Michigan | USA | 8 | 258 | 32 | 8 |
| 3 | The University of Hong Kong | China | 8 | 26 | 3 | 3 |
| 4 | Zhengzhou University | China | 7 | 53 | 8 | 10 |
| 5 | Icahn School of Medicine at Mount Sinai | USA | 7 | 199 | 28 | 5 |
| 6 | Southern Medical University | China | 7 | 160 | 23 | 5 |
| 7 | China Academy of Chinese Medical Sciences | China | 7 | 68 | 10 | 3 |
| 8 | Wuhan University | China | 7 | 38 | 5 | 2 |
| 9 | Sun Yat-sen University | China | 6 | 23 | 4 | 7 |
| 10 | Jinan University | China | 6 | 87 | 15 | 5 |

Abbreviations: USA= United States of America
