# Supplementary material for: Bibliometrics and visualization analysis: Big data research on diabetic kidney disease from 2000 to 2025
Source: Medicine (Baltimore). 2026 Aug 14;105(33):e50234. doi: 10.1097/MD.0000000000050234 (PMC13480767; doi:10.1097/MD.0000000000050234)
Supplement: Supplementary file 3 [file medi-105-e50234-s003.docx]

**Supplementary Table 3.** The top 10 citations of authors.

| Rank | Author | Organizations | Citations | Documents | Average citation | Total link strength |
| --- | --- | --- | --- | --- | --- | --- |
| 1 | Kretzler, Matthias | University of Michigan | 318 | 5 | 64 | 16 |
| 2 | Sarder, Pinaki | University of Florida | 252 | 5 | 50 | 21 |
| 3 | Ju, Wenjun | University of Michigan | 251 | 5 | 50 | 16 |
| 4 | Jain, Sanjay | Washington University School of Medicine | 235 | 3 | 78 | 13 |
| 5 | Bitzer, Markus | University of Michigan | 214 | 4 | 54 | 16 |
| 6 | Nair, Viji | University of Michigan | 213 | 3 | 71 | 14 |
| 7 | Hodgin,Jeffrey b. | University of Michigan | 205 | 3 | 68 | **12** |
| 8 | Tomaszewski, John E. | University at Buffalo | 199 | 4 | 50 | 17 |
| 9 | Yacoub, Rabi | Icahn School of Medicine at Mount Sinai | 199 | 3 | 66 | 14 |
| 10 | Jen, Kuang-yu | University of California | 194 | 3 | 65 | 15 |
